# Complete Genome Sequence of Campylobacter hepaticus USA52, Associated with Chicken Spotty Liver Disease

**DOI:** 10.1128/MRA.01266-20

**Published:** 2021-02-25

**Authors:** Zuowei Wu, Orhan Sahin, Qijing Zhang

**Affiliations:** a Veterinary Microbiology and Preventive Medicine, Iowa State University, Ames, Iowa, USA; b Veterinary Diagnostic and Production Animal Medicine, Iowa State University, Ames, Iowa, USA; University of Maryland School of Medicine

## Abstract

Campylobacter hepaticus was recently identified as the etiological agent of chicken spotty liver disease. Here, we report the complete genome sequence of *C. hepaticus* strain USA52 from the United States. The genome comprises a chromosome of 1,509,100 bp with an average GC content of 28.02%.

## ANNOUNCEMENT

Spotty liver disease (SLD) is an acute infectious disease of layer poultry and has become an important concern for the poultry egg and meat industries (1–3). Although the disease was originally reported in the United States and Canada in the 1950s, the definitive etiological agent was not identified until 2015, when a new *Campylobacter* species, C. hepaticus, was determined to be the causative agent in the United Kingdom and Australia (4–6). In the United States, *C. hepaticus* was first identified from SLD layers in 2017 (7). However, to date, a complete genome sequence of *C. hepaticus* from the United States has not been reported. Here, we report the complete genome sequence of *C. hepaticus* strain USA52.

*C. hepaticus* USA52 was isolated from the bile of a dead SLD layer as described previously (4). The bile sample was submitted to our diagnostic lab by a farm with an SLD outbreak in Nebraska. Strain USA52 was identified to be *C. hepaticus* by 16S rRNA gene sequencing and PCR detection of the glycerol kinase gene as described previously (7). Strain USA52 cells were collected after culturing on blood agar plates (Remel, Lenexa, KS) for 3 days at 37°C, and genomic DNA was extracted using a Genomic-tip 20/G kit (Qiagen, Germantown, MD). A Nanopore library was constructed with 1.5 μg of genomic DNA using the Nanopore ligation sequencing kit (SQK-LSK108) (Oxford Nanopore Technologies, Cambridge, MA). Short DNA fragments (<10 kb) were removed from the library using a short-read eliminator (SRE) XS kit (Circulomics, Baltimore, MD). The library was sequenced on the Oxford Nanopore GridION X5 sequencing platform (flow cell, FLO-MIN106), and the reads were base called using Guppy v3.2.8 (8) at Iowa State University. The library yielded 4,000 reads with an *N*_50_ value of 19,540 bp, providing 394-fold coverage for the complete genome sequence. An Illumina short-read library was prepared with 1 μg of the same genomic DNA extract as above using the NEBNext Ultra II DNA FS library kit (New England Biolabs, Ipswich, MA) and sequenced on the Illumina MiSeq platform in 150-bp paired-end mode at Iowa State University, yielding 99-fold genome coverage with 1,003,036 reads. The read quality was examined using FastQC v0.11.9 (9). The Nanopore reads were *de novo* assembled using Flye v2.4 (10), producing one 1,559,990-bp contig. The contig was then error corrected with the Illumina reads using Pilon v1.23 (11). The corrected contig was circularized into a chromosome of 1,509,100 bp with a GC content of 28.02% by removing the overlapping ends manually after aligning it using Mauve v2.13 (12), with the contigs (assembled by Velvet v1.2.10 [13]) from the Illumina reads. The genes were annotated using the NCBI Prokaryotic Genome Annotation Pipeline (PGAP) v4.13 (14). The chromosome sequence was rotated to start at the *dnaA* gene using GAMOLA2 (15). Default parameters were applied for all software. The chromosome contained 1,484 predicted protein-coding sequences (CDSs), 3 sets of rRNA genes, 3 noncoding RNA (ncRNA) genes, and 43 tRNA genes.

No reviews from the Institutional Ethics Committee and the Institutional Animal Care and Use Committee were required, because this study did not include any animal studies.

### Data availability.

The complete genome sequence of *C. hepaticus* USA52 has been deposited at GenBank under accession number CP063536. The raw sequence data have been deposited at the GenBank Sequence Read Archive under accession numbers SRR12900734 (Illumina reads) and SRR12900733 (Nanopore reads).
